# A Multi-Sensorial Simultaneous Localization and Mapping (SLAM) System for Low-Cost Micro Aerial Vehicles in GPS-Denied Environments

**DOI:** 10.3390/s17040802

**Published:** 2017-04-08

**Authors:** Elena López, Sergio García, Rafael Barea, Luis M. Bergasa, Eduardo J. Molinos, Roberto Arroyo, Eduardo Romera, Samuel Pardo

**Affiliations:** Electronics Department, University of Alcalá, Campus Universitario, 28805 Alcalá de Henares, Spain; sergio.garciagonzalo@edu.uah.es (S.G.); rafael.barea@uah.es (R.B.); luism.bergasa@uah.es (L.M.B.); eduardo.molinos@edu.uah.es (E.J.M.); roberto.arroyo@edu.uah.es (R.A.); eduardo.romera@edu.uah.es (E.R.); samuel.pardoa@edu.uah.es (S.P.)

**Keywords:** aerial robots, SLAM, sensor fusion

## Abstract

One of the main challenges of aerial robots navigation in indoor or GPS-denied environments is position estimation using only the available onboard sensors. This paper presents a Simultaneous Localization and Mapping (SLAM) system that remotely calculates the pose and environment map of different low-cost commercial aerial platforms, whose onboard computing capacity is usually limited. The proposed system adapts to the sensory configuration of the aerial robot, by integrating different state-of-the art SLAM methods based on vision, laser and/or inertial measurements using an Extended Kalman Filter (EKF). To do this, a minimum onboard sensory configuration is supposed, consisting of a monocular camera, an Inertial Measurement Unit (IMU) and an altimeter. It allows to improve the results of well-known monocular visual SLAM methods (LSD-SLAM and ORB-SLAM are tested and compared in this work) by solving scale ambiguity and providing additional information to the EKF. When payload and computational capabilities permit, a 2D laser sensor can be easily incorporated to the SLAM system, obtaining a local 2.5D map and a footprint estimation of the robot position that improves the 6D pose estimation through the EKF. We present some experimental results with two different commercial platforms, and validate the system by applying it to their position control.

## 1. Introduction

Research on autonomous aerial robots has advanced considerably in the last decades, especially in outdoor applications. Enabled by MEMS inertial sensors and GPS, Unmanned Aerial Vehicles (UAVs) that show an awesome set of flying capabilities in outdoor environments have been developed, ranging from typical flight manoeuvres [1], to collaborative construction tasks [2] or swarm coordination [3], among many other applications.

Although technological progress has made possible the development of small Micro Aerial Vehicles (MAVs) capable of operating in confined spaces, indoor navigation is still an important challenge for a number of reasons: (i) most indoor environments remain without access to external positioning systems such as GPS; (ii) the onboard computational power is restricted and (iii) the low payload capacity limits the type and number of sensors that can be used. However, there is a growing interest in indoor applications such as surveillance, disaster relief or rescue in GPS-denied environments (such as demolished or semi-collapsed buildings). In these stages is often preferable to use low-cost MAVs that can be easily replaced in case of breakage, damage or total loss.

From the navigation point of view, state estimation of the six degrees of freedom (6-DoF) of the MAV (attitude and position) is the main challenge that must be tackled to achieve autonomy. The inaccuracy and high drift of MEMS inertial sensors, the limited payload for computation and sensing, and the unstable and fast dynamics of air vehicles are the major difficulties for position estimation. So far, the most robust solutions are based on external sensors, as in [4], where an external trajectometry system directly yields the position and orientation of the robot, or in [5], where an external CCD camera provides the measurements. However, these solutions require a previous preparation of the environment and are not applicable to unknown spaces, in which the MAV must rely on its own onboard sensors to navigate.

The problem of building a map of an unknown environment from onboard sensor data while simultaneously using the data to estimate the robot’s position is known as Simultaneous Localization and Mapping (SLAM) [6]. Robot sensors have a large impact on the algorithm used in SLAM. In the last ten years, a large amount of research has been devoted to 2D laser range finders [7,8], 3D lidars [9,10] and vision sensors [11,12]. Recent research pay attention to many other alternatives that can be leveraged for SLAM, such as light-emitting depth cameras [13], light-field cameras [14], and event-based cameras [15], as well as magnetic [16], olfaction [17] and thermal sensors [18]. However, these alternative sensors have not yet been considered in the same depth as range and vision sensors to perform SLAM.

Although there have been significant advances in developing accurate and drift-free SLAM algorithms for ground robots using different sensors and algorithms [6,19], attempts to achieve similar results with MAVs have not been as successful. MAVs face a number of specific challenges that make developing algorithms for them much more difficult: limited sensing payload and onboard computation, indirect relative position estimation, fast dynamic, need to estimate velocity and constant motion, among others.

In aerial navigation, sensors must be carefully chosen due to mobility, design and payload limitations. All aerial robots are endowed with an Inertial Measurement Unit (IMU) that combines accelerometers, gyroscopes and sometimes magnetometers to measure orientation, angular rate and accelerations. However, tracking the MAV position using dead reckoning based on the IMU (which is known as Inertial Navigation System—INS) produces large errors and drifts [20], so the solution always involves fusing these measurements with other extereoceptive sensors. When GPS is available, the fusion of IMU and GPS information results in INS/GPS systems that yield satisfactory results in outdoor environments [21]. Nevertheless, in GPS-denied environments other alternatives should be explored.

Due to their low weight and consumption, most commercial MAVs incorporate at least one monocular camera, so Visual SLAM (VSLAM) methods have been widely used [22,23,24,25]. However, most of these works have been limited to small workspaces with definite image features and well lit, and thus may not work as well for general navigation in GPS-denied environments. In addition, computational time is too high for the fast dynamics of aerial robots, making difficult to control them. Furthermore, despite their greater consumption and weight, range sensors such as RGB-D cameras [26,27] or 2D range sensors [28] have also been used on MAVs due to their direct distance detection. Especially, the high working rate of current 2D laser scanners, along with their direct and accurate range detection, make them a very advantageous sensor for indoor aerial navigation. Several works, such as [29,30], fuse laser an IMU measurements to obtain 2D maps and to estimate the 6-DoF pose of the MAV.

In this paper, we propose a SLAM system for GPS-denied environments that can be easily configured according to the onboard sensors of low-cost MAVs. This work is motivated by a recent research line of the RobeSafe Research Group [31] of the University of Alcalá (Spain), whose final aim is the development of a heterogeneous swarm of robots for disaster relief. The swarm consists of an Unmanned Ground Vehicle (UGV) and one or more MAVs for accessing difficult areas. Low-cost MAVs and their on-board sensors have been chosen in order to be easily replaced in the event of breakage or loss. Besides, the SLAM system is executed by a remote PC onboard the UGV, offsetting the low onboard computing capacity of MAVs and facilitating their replacement.

This paper focuses on the development of the SLAM system for the MAVs based on IMU, vision and laser sensors (depending on their availability) and fusing different state-of-the-art SLAM methods. The system robustly obtains the 6-DoF pose of the MAV within a local map of the environment. We consider a minimum sensory configuration based on a frontal monocular camera, an IMU and an altimeter. Two recent and robust monocular VSLAM techniques (LSD-SLAM [32] and ORB-SLAM [33]) have been tested and compared when applied to aerial robots, whose onboard sensors permit to solve scale ambiguity and to improve pose estimation through an Extended Kalman Filter (EKF). When available, a 2D laser sensor is used to obtain a local 2.5D map and a footprint estimation of the pose of the MAV, that improve pose estimation in environments with few visual features and/or in low light conditions. The system has been validated with two low-cost commercial drones with different sensor configurations and a remote control unit using a distributed node system based on Robot Operating System (ROS) [34]. The experimental results show that sensor fusion improves position estimation and the obtained map under different test conditions.

The paper is organized as follows: Section 2 describes the overall system, including hardware platforms used and software architecture. The SLAM approach is explained in detail in Section 3. Section 4 presents the experimental results. Finally, in Section 5 and Section 6 the discussion and conclusion of this work are presented.

## 2. System Overview

We face the problem of autonomous MAV state estimation as a software challenge, focusing on high-level algorithms integration rather than specific hardware. Some state-of-the-art SLAM methods have been tested, compared and fused under different sensor configurations and environment conditions, allowing to obtain conclusions about their capability to be applied to aerial robots. For this reason, we use low-cost commercial platforms and an open-source development environment (ROS), so that drivers of sensors and some algorithms can be used without development.

### 2.1. Assumptions and Notation

**Figure 1:**
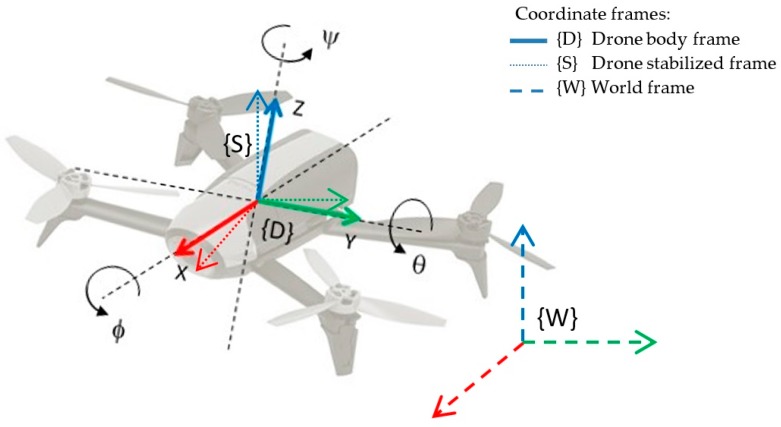
Coordinate frames {D}, {S} and {W}: drone frame {D} is attached to the drone’s body; stabilized frame {S} is parallel to the floor; world frame {W} matches the initial position of the drone.

We consider a quadrotor freely moving in any direction in ℜ3 × SO(3). The three main coordinate frames considered are: (1) the drone coordinate frame {D} attached to its body; (2) the drone stabilized frame {S}, whose attitude (roll/pitch angles) are corrected to be parallel to the floor; and (3) the world coordinate frame {W}, that matches the initial position of the drone and is always at ground level. All the coordinate systems are right handed defined, as shown in Figure 1. Orientation is specified in Euler XYZ (roll-pitch-yaw) angles notation, (φ,θ,ѱ).

Throughout this document, the following notation is used to distinguish actual, measured and commanded variables: the actual variable is indicated without emphasis, while the corresponding measured variable is emphasized with “–”accent, and the commanded one with “^” accent. For example, $\hat{vz}$ is the commanded vertical velocity, while $\bar{vz}$ and vz are the measured and the actual vertical velocities respectively.

### 2.2. Hardware Platforms

One of the main specifications of the proposed SLAM system is that it can be configured to be implemented with different low-cost MAVs, using their onboard sensors. Usually, the software running onboard a commercial MAV is not accessible, and the control software is neither open-source nor documented in any way. Indeed, many low-cost MAVs are primarily sold as high-tech toys and only can be commanded from their own applications running on a Smartphone or a laptop. However, other platforms provide a Software Development Kit (SDK) for programming applications by sending commands through an ad-hoc wireless LAN network set up by the drone, opening the door to research works. Some of these MAVs, whose drivers are available as ROS packages to be easily used, are the Pelican and Hummingbird quadrotors of Ascending Technologies [35], the CrazyFlie of Bitcraze [36], the Matrice 100 of DJI [37], the Erle-copter of Erle Robotics [38], or the AR-Drone and Bebop drones of Parrot [39].

On the other hand, most commercial MAVs incorporate an IMU, a sensor for measuring height (ultrasound and/or barometer) and at least one monocular horizontal camera. It conforms the minimal onboard sensor configuration required by our proposed SLAM system. Some drones with a limited onboard computational capacity and a greater payload can easily incorporate a light 2D laser rangefinder, which will be considered as an additional sensor that will greatly improve the results of the SLAM system under certain environment conditions.

**Figure 2:**
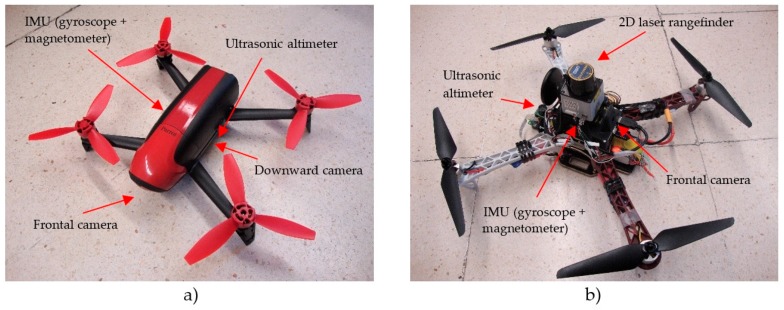
Commercial low-cost platforms used for our experiments, with the required minimal onboard configuration: (**a**) Bebop Drone of Parrot; (**b**) Erle-Copter of Erle Robotics.

For our experiments, we have chosen two commercial platforms that meet the following requirements: (1) low-cost and small dimensions; (2) minimal onboard sensory configuration consisting of IMU, altimeter and frontal camera; and (3) SDK for programming applications by sending commands and reading sensors through a wireless local network (and in both cases, with driver available in ROS framework). These platforms, shown in Figure 2, are:

1. The Parrot Bebop (Parrot, Paris, France). This is a light (400 g) and small (33 × 38 × 3.6 cm) drone, ideal for indoor applications. It is equipped with a frontal “Fisheye” camera, and another vertical camera, which is internally used for stabilization and horizontal velocity estimation. Besides, it has an ultrasonic altimeter, a 3-axis accelerometer, two gyroscopes and a barometer. It incorporates an onboard controller (dual-core Parrot P7 processor), a quad-core graphic processor, flash memory of 8 Gb and a Linux distribution. ROS framework provides the *bebop_autonomy* package [40] as a driver for communicating with the drone through its wireless local network.
2. The Erle-Copter (Erle Robotics, Vitoria, Spain). This drone weights 1.3 kg and its size is 36 × 34 × 9.5 cm. It has a greater payload than Bebop (1 kg) and an onboard open-brain (ErleBrain2) based on a Raspberry PI with the ROS framework and the APM autopilot. This allows to board a light 2D laser sensor such as the Hokuyo URG-04LX, and we have added the *hokuyo_node* ROS package [41] to easily read and transmit the laser measurements to the remote processor. The Erle-Copter can be accessed and commanded from ROS using the *mavros* package [42].

It is important to remark that the proposed SLAM approach can be applied to other similar platforms and so we treat the drone as a black box, using only the available W-LAN communication channels to access and control it. Namely, the following inputs/outputs are used in our SLAM system:

- A command output channel, to send the drone control packages ***u*** with the desired velocities of *x* and *y* axis, vertical speed and yaw rotational velocity, all them defined respect to the drone stabilized frame {S}:
  (1) $$u=\left(\hat{vx},\hat{vy},\hat{vz},\hat{\dot{\psi }}\right)$$
- A video input channel, to receive the video stream of the forward facing camera.
- A navigation input channel, to read onboard sensor measurements. The minimum data required by our system are:
  - Drone orientation as roll, pitch and yaw angles $\left(\bar{\phi },\bar{\theta },\bar{\psi }\right)$ respect to the world frame {W}.
  - Drone height $\bar{h}$, obtained from the altimeter.

Other measurements can be incorporated to the SLAM system when available. For example, the Bebop drone incorporates a downward camera that, by means of an optical-flow based motion estimation algorithm calculated onboard, allows relative precise estimated horizontal velocities in the stabilized coordinate frame {S}, $\left(\bar{vx},\;\bar{vy}\right)$. This measurement can be easily incorporated through the Extended Kalman Filter, as will be shown in a future section. Even more importantly, the availability of an onboard 2D laser rangefinder, as in the case of the Erle-copter (Figure 2b), allows to introduce these measurements to a scan matcher module of the SLAM system that will notably enhance the estimation results.

### 2.3. Software Architecture

**Figure 3:**
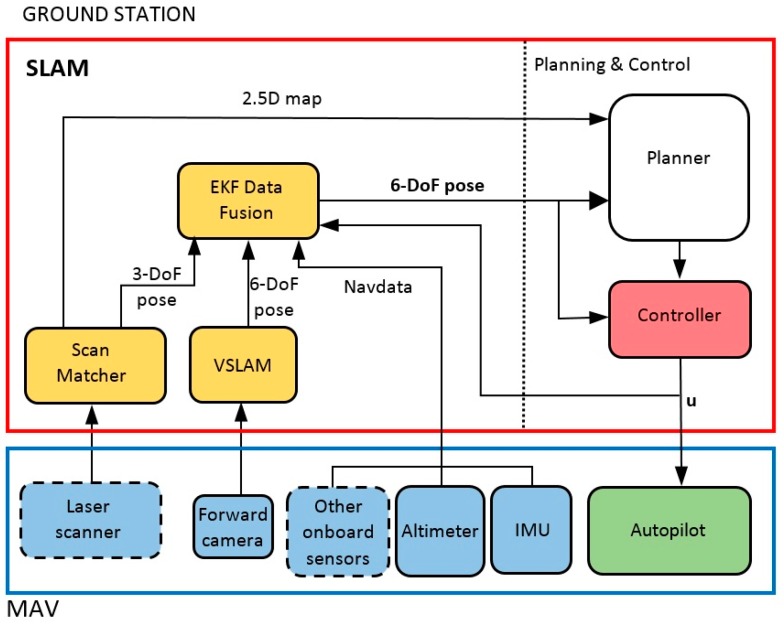
Software architecture. The onboard computer of the MAV: (1) reads sensors (blue blocks with continuous line correspond to minimum sensory configuration and blue blocks with dashed line to optional sensors) and (2) receives commands for the autopilot (green block). The ground station executes the SLAM system (yellow blocks), the controller (red block) and the planner, that is out of the scope of this paper.

As stated above, the developed system is distributed between the flying unit (MAV) and a remote ground station, as it is shown in Figure 3. The flying unit is treated as a black box, and includes at least one onboard controller/processor that executes an autopilot and performs the reading and sending of onboard sensors measurements. When a laser sensor is present, as in the Erle-Copter, it is connected to the onboard processor that needs to be able to read and send these measurements through the network created by the drone. The SLAM module is executed in the ground station, as well as the control and planning modules, the last one being out of the scope of this paper. The use of ROS as development framework facilitates the setting of this distributed system.

The SLAM system consist of three major modules [43]: (1) a scan matching algorithm that uses laser readings to obtain a 2.5D map of the environment and a 3-DoF pose estimation of the footprint of the MAV on the map; (2) a monocular visual SLAM system that obtains a 6-DoF pose estimation and (3) an Extended Kalman Filter that fuses the last estimations with the navigation data provided by the onboard sensors of the MAV to obtain a robust 6-DoF estimation of the position of the robot.

In order to test the SLAM system in autonomous flying conditions, a simple PID controller has been developed. This controller allows the MAV to reach selected reference poses in order to autonomously track a desired path.

## 3. SLAM Method Description

In the following subsections, we describe the modules of the SLAM system shown in Figure 3. For the scan matcher and VSLAM modules, we analyze and compare state-of-the-art techniques that can be applied to aerial robots. The estimates of these modules are integrated with the rest of onboard sensors using the EKF module.

### 3.1. Monocular Visual SLAM

In order to perform the simultaneous localization and mapping of the environment by means of visual information, the process of Visual Odometry (VO) must be accomplished. VO is the process of determining the position and orientation of a robot by analyzing the associated camera images, thus, to estimate the 6-DoF position of the MAV. The VO approaches can be classified into two main categories based on the number of cameras used [44]: monocular and stereo VO methods. A stereo pair is applied as minimum number configuration of cameras for solving the scale ambiguity problem [45]. So, monocular VO methods used for single-camera MAVs have the implicit problem of scale ambiguity. In this work, a method for calculating the scale of the estimated pose and map based on additional MAV sensors is proposed.

On the other hand, monocular VSLAM methods that simultaneously recover camera pose and scene structure from video can be divided into two classes [46]: (a) feature-based methods, that firstly extract a set of feature observations from the image, and then compute the camera position and scene geometry as a function of these feature observations and (b) direct methods (dense or semi-dense), that optimize the geometry directly on the image’s pixels intensities, which enables using all information in the image.

**Figure 4:**
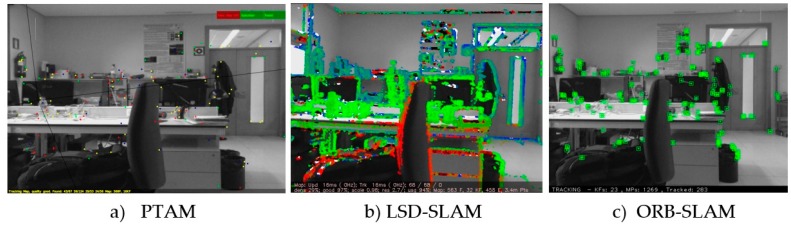
Image processing using some of the most significant monocular VSLAM methods: (**a**) PTAM; (**b**) LSD-SLAM and (**c**) ORB-SLAM.

Most monocular VO algorithms for MAVs [47,48,49] rely on PTAM [50]. PTAM is a feature-based VSLAM algorithm that achieves robustness through tracking and mapping many (hundreds) of features (Figure 4a). It runs in real-time by parallelizing the mapping and motion estimation tasks. However, PTAM was designed for augmented reality applications in small desktop scenes and it does not work properly in large-scale environments.

Recently, more robust and efficient monocular VSLAM methods have been proposed in the literature [51]. In [52], a semi-direct monocular visual odometry algorithm, Semi-direct Visual Odometry (SVO) is presented. This algorithm has been successfully applied to MAVs with a downfacing camera and outputs a sparse 3D reconstructed environment model, but it is not designed to work with forward facing cameras. In [32], the authors describe a direct monocular VLAM algorithm for building consistent, semi-dense reconstructions of the environments, the LSD-SLAM method (Figure 4b). LSD SLAM employs a pose graph optimization which explicitly allows for scale drift correction and loop closure detection in real-time. LSD SLAM employs three parallel threads after initialization takes place: tracking, depth map estimation, and map optimization. A modified version of LSD-SLAM was later presented in [53] for a stereo camera setup. Later, Dense Piecewise Parallel tracking and Mapping (DPPTAM) [54], was released as a semi-dense direct method similar to LSD-SLAM but including a new thread that performs dense reconstructions using segmented super-pixels from indoor planar scenes. Finally, in [33], a keyframe-based monocular VSLAM system with ORB features that can estimate the 6-DoF pose and reconstruct a sparse environment model is presented (ORB-SLAM—Figure 4c). The main contributions of ORB-SLAM are the usage of ORB features in real-time, re-localization with invariance to viewpoint and a place recognition module that uses bags of words to detect loops.

After a study of these state-of-the-art monocular VSLAM methods [51], we decided to implement and compare two of these algorithms in our system [55]: LSD-SLAM and ORB-SLAM, both available as ROS packages and with good execution-times to be implemented on MAVs. Scale ambiguity problem, implicit in both monocular methods, has been overcomed by using measurements of other onboard MAV’s sensors and comparing them with the VSLAM ones. Taking into account that scale factor is different for x, y and z axis, the best solution is to use the altimeter for estimating the z-axis scale, and the laser sensor, when available, to estimate x-axis and y-axis scale as follows:

(2) $$scale_{z}=\frac{h_{altimeter}}{h_{VSLAM}};\;scale_{x}=\frac{dx_{laser}}{dx_{VSLAM}};\;scale_{y}=\frac{dy_{laser}}{dy_{VSLAM}};$$

(3) $$x_{real-scale}=x_{VSLAM}\cdot scale_{x}$$

(4) $$y_{real-scale}=y_{VSLAM}\cdot scale_{y}$$

(5) $$z_{real-scale}=z_{VSLAM}\cdot scale_{z}$$

where *h_altimeter_* is the direct altimeter measurement, *dx_laser_* and *dy_laser_* are the laser range measurements in *x* and *y* directions, and *h_VSLAM_*, *dx_VSLAM_* and *dy_VSLAM_* are the equivalent estimates of the VSLAM system. As *h_VSLAM_* = *z_VSLAM_*, it can be seen that *z_real-sale_* is directly the altimeter measurement, that is very accurate. If laser is not available, we suppose the same scale factor for the three axis, and estimate it using the altimeter. The error committed under this assumption is negligible in narrow environments, as the indoor ones to which our SLAM system has been designed. However, it could be increased in wider outdoor environments. The scale factors are updated at each iteration of the SLAM system, because they may change over time.

In the following subsections, we show some partial results of the VSLAM module with scale factor correction using the two chosen algorithms, so that some conclusions can be obtained about their performance when applied to aerial robots.

#### 3.1.1. VSLAM with LSD-SLAM

LSD-SLAM performs a highly accurate pose estimation from direct image alignment, and obtains in real-time a 3D reconstruction of the environment as pose-graph of keyframes with associated semi-dense depth maps. We use these maps as local information for VO but, due to their computational and memory requirements, we discard these dense maps for reconstructing the environment. When laser is available, we chose to use the 2.5D laser map for planning and control because its better accuracy and lower computational requirements are more appropriate to control a fast dynamic plant as a MAV.

**Figure 5:**
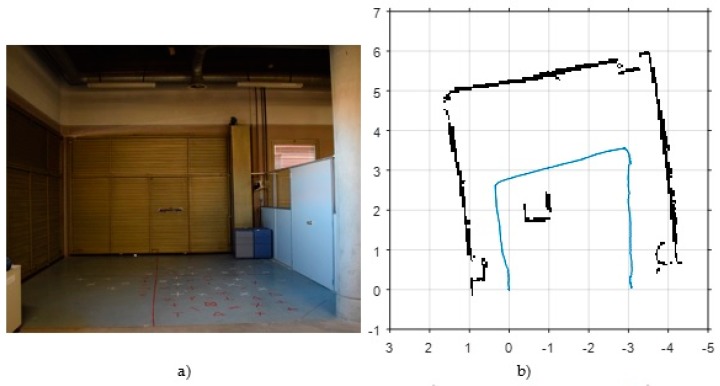

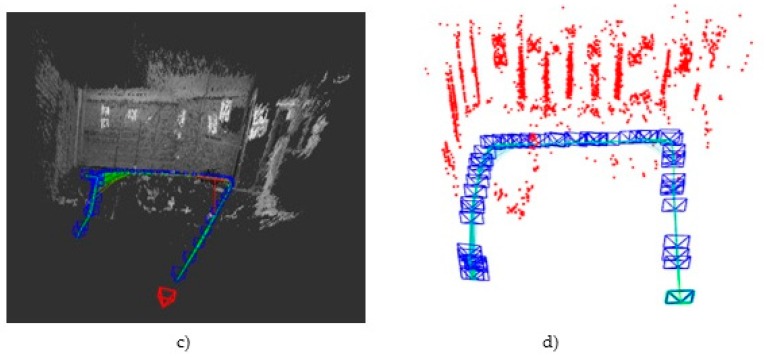
Initial results of state-of-the art SLAM algorithms in a storage area (poorly lit environment). (**a**) View of the test environment; (**b**) Results of “Hector-mapping”: black points represent the 2D map and the blue line shows the estimated trajectory; (**c**) Results of “LSD-SLAM” with scale correction: grey points conform the semi-dense map, the green line represents the estimated trajectory and the blue marks are the camera’s poses where a new keyframe was captured; (**d**) Results of “ORB-SLAM” with scale correction: the sparse map of ORB-features is coloured in red, the green line represents the estimated trajectory and the blue marks are the camera’s poses where a new keyframe was captured.

**Figure 6:**
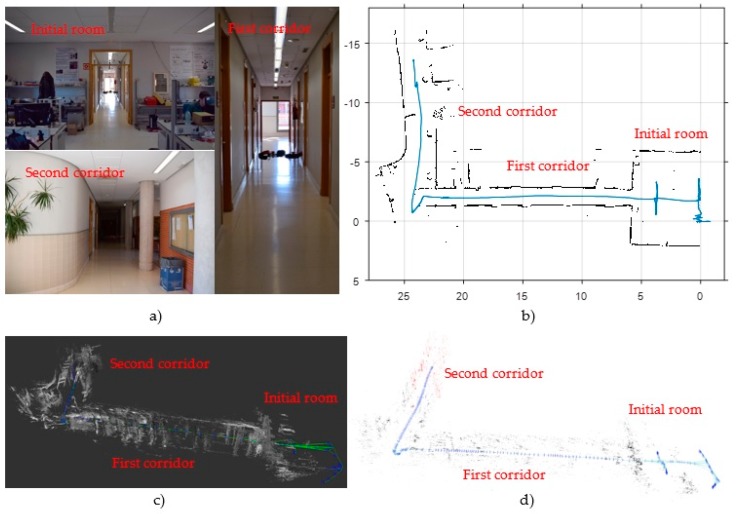
Initial results of state-of-the-art SLAM algorithms in a corridor area (a poorly featured environment). (**a**) View of the test environment; (**b**) “Hector-mapping” results: black points represent the 2D map and the blue line shows the estimated trajectory ; (**c**) “LSD-SLAM” results with scale correction: grey points conform the semi-dense map, the green line represents the estimated trajectory and the blue marks are the camera’s poses where a new keyframe was captured; (**d**) “ORB-SLAM” results with scale correction: the sparse map of ORB-features is coloured in red, the green line represents the estimated trajectory and the blue marks are the camera’s poses where a new keyframe was captured.

In order to make an initial assessment about the performance of LSD-SLAM in visually adverse scenarios, we have chosen two test environments. The first one, shown in Figure 5a, is a storage area of our building with poor lighting. Figure 6a shows a view of the second scenario, that consists of a room followed by two L-shaped corridors. Corridors are visually unfavourable scenarios due to the lack of features. Figure 5c and Figure 6c show the results for both scenarios, consisting in the 3D semi-dense map and pose estimation obtained by the LSD-SLAM technique with scale correction.

As a coarse evaluation, and using some metrics of the actual MAV trajectory, we obtain the following undesirable effects: (1) in the storage area, the lack of light causes a clear lengthening in the estimation of the second segment of the trajectory, from 4 to 5.5 m approximately; (2) by contrast, in the corridor environment the pose estimation is highly accurate in the initial room, but a clear shortening effect of around 25% is observed in the corridors (from 22 m to 16 m in the first corridor and from 16 to 12 m in the second one), due to the loss of visual features. Furthermore, LSD-SLAM is very sensitive to pure rotational movements as the one at the end of the first corridor, where an error of about 15° is observed.

#### 3.1.2. VSLAM with ORB-SLAM

Figure 5d and Figure 6d show the initial results obtained with ORB-SLAM with scale correction in the same test environments. They show in red colour the ORB-features map and in green colour the estimated trajectory. As a featured-based method, the greatest weakness of ORB-SLAM lies again in poorly featured areas as corridors. In this case, corridors are also shortened about a 35%, from 22 to 14 m the first one, and from 16 to 10 m the second one. However, this method is more accurate than LSD-SLAM in well-lit rooms and in the storage room scenario. In the latter case, the second segment is also lengthened due to poor illumination, but in this case only from 4 to 4.4 m. Besides, ORB-SLAM is more precise than LSD-SLAM in pure rotational movements.

#### 3.1.3. Comparison

In order to have a clearer comparative of both algorithms in terms of accuracy, an external public benchmark has been applied. The chosen benchmark is “RGB-D SLAM Dataset and Benchmark” [56] of the Computer Vision Group from Technische Universität München (TUM). This benchmark provides some datasets with measurements from different sensors and the ground-truth pose of the camera. As output, it delivers a variety of measures of translational errors. For our tests, the dataset *rgbd_dataset_freiburg1_xyz* has been used. This dataset contains a video recorded from a monocular camera that describes smooth and rotation free movements that are perfect for an initial comparison of translational errors. In the results section of this paper a more thorough analysis is performed with our own datasets and benchmark.

Table 1 shows the median of the results obtained when executing the two VSLAM algorithms five times each with the mentioned dataset. As the real scale cannot be calculated by the monocular algorithms by themselves, the estimations extracted from the dataset were pre-processed. Thanks to it the real-scale was calculated with a Matlab script and added as an argument in the online tool. Each of the methods has a different number of compared pairs because not all of the estimated poses are compared, but only those whose timestamps match the given by the ground truth.

According with these results, ORB-SLAM is slightly more accurate than LSD-SLAM in terms of translational errors. In addition, ORB-SLAM is more robust facing pure rotational movements. This conclusion has been reached by means of the trial-and-error approach (LSD-SLAM loses the tracking more times than ORB-SLAM when the camera suffers pure rotational movements). However, the dataset used has many visual features to extract, which benefits feature-based methods as ORB-SLAM. LSD-SLAM, as direct method, achieves better results in poorly featured environments. Besides, the initialization of LSD-SLAM is self-acting from initial random values given to the depth map, while ORB-SLAM needs a specific initialization stage to build a points map of the environment before starting the tracking.

As the SLAM system has been designed to execute the VSLAM module in a ground station (in our tests an Intel Core i7-3635QM, 2.4 GHz), these execution times (with median speeds of 36.2 ms for LSD-SLAM and 32.2 ms for ORB-SLAM) are both feasible for tracking the fast dynamics of a MAV.

### 3.2. Scan Matcher

When a 2D laser rangefinder is available on the MAV, it can be used to substantially improve the pose and map estimation under certain environment conditions. It is clear that VSLAM methods cannot work in low light conditions and their results are poor in featureless environments such as corridors. By contrast, laser sensors work properly under all light conditions and provide high-frequency and direct range measurements of the environment. So, this sensor not only provides redundancy, but also complementarity, and can be easily included in the bayesian framework of our SLAM system through the Extended Kalman Filter, as it is shown in Figure 3.

The Scan Matcher module aligns consecutive scans from a laser to improve the MAV’s motion estimation of the EKF. Although lots of scan matching techniques have been developed and applied to SLAM for ground robots moving on flat surfaces [57], most of them require odometric information that is not available in MAVs. One of the simplest and most commonly used algorithm for laser scan-matching is Iterative Closest Point (ICP) [58]. The main drawback of most ICP-based methods is the expensive search for point correspondences, which has to be done at each iteration. As a solution, methods based on feature-to-feature correspondences [59] or likelihood maps [60] have also been proposed. One of the benefits of the last ones is that they do not require explicit correspondences to be computed, and can be easily integrated into probabilistic SLAM techniques. Thus, one of the most reliable working solutions for laser-based SLAM is Gmapping [57], based on likelihood maps and Rao-Blackwellized particle filters. This algorithm is available as open source software, and provides successful results in ground robots moving in typical planar indoor scenarios. However, it relies on sufficiently accurate odometry (not available in aerial robots) and is not applicable to platforms with significant roll and pitch motion. However, the HectorSLAM project, developed by the Team Hector [61] of the Technische Universität Darmstadt, presents a system for fast online generation of 2D maps that uses only laser measurements and a 3D attitude estimation system based on inertial sensing [62]. This method requires low computational resources and it is widely used by different research groups because of its availability as open source based on ROS. More recent methods have been suggested in the last years, such as the one proposed in [8], an initial-free 2D laser scan matching method based on point and line features detection. This method, however, requires a pre-processing stage to detect features from scans that reduces the working rate and is not optimal for fast systems as a MAV.

In this work, we adapt the HectorSLAM system to our scan matching module. It allows the system to obtain a 2.5D map and a 3-DoF estimation of the MAV’s footprint pose within the map, consisting in the (x,y) coordinates and yaw angle ѱ in the world coordinate frame {W}.

The 3-DoF pose estimation is based on optimization of the alignment of beam endpoints with the map obtained so far [62]. The endpoints are projected into the current map and the occupancy probabilities are estimated. The scan matching is solved using a Gaussian-Newton equation, which finds the rigid transformation that best fits the laser beams with the map. A multi-resolution map representation is used to avoid getting stuck in local minima. In addition, an attitude estimation system based on the IMU measurements transforms the laser readings from the drone body frame {D} to the drone stabilized frame {S} (horizontal to the ground, as can be seen in Figure 1) in order to compensate the roll and pitch movements when obtaining the 2.5D map.

Figure 5b and Figure 6b show the map and footprint pose estimation obtained by the scan matcher in the same environments of VSLAM. Although HectorSLAM provides good results in confined environments (Figure 5b), the lack of odometry information to detect horizontal movements (the only measurement used by the algorithm from the IMU is attitude, in order to stabilize laser measurements into the {S} coordinate frame) results in undesirable effects, such as shortening of corridors (from 22 m to 20 m in the first corridor of Figure 6b).

These drawbacks will be compensated in our whole proposed SLAM system due to the addition of visual and other onboard sensors information, as well as prediction estimates, as will be shown in the results section.

### 3.3. Data Fusion with EKF

In order to fuse all available data, we use an EKF [63]. This EKF is also employed to compensate for the different time delays in the system, arising from wireless LAN communication and computationally complex visual processing. The proposed EKF uses the following state vector:

(6) $$\chi_{t}:{\left(x_{t},y_{t},z_{t},vx_{t},vy_{t},vz_{t},\phi_{t},\theta_{t},\psi_{t},{\dot{\psi }}_{t}\right)}^{T}\;\in \;{ℜ}^{10}$$

where $\left(x_{t},y_{t},z_{t}\right)$ is the position of the MAV in m, $\left(vx_{t},vy_{t},vz_{t}\right)$ the velocity in m/s, $\left(\phi_{t},\theta_{t},\psi_{t}\right)$ the roll, pitch and yaw angles in degrees, and ${\dot{\psi }}_{t}$ the yaw-rotational speed in deg/s, all of them in world coordinate frame {W}. In the following sections, we define the prediction and observation models.

#### 3.3.1. Prediction Model

The prediction model is obtained from the full motion model of the quadcopter’s flight dynamics and reaction to control commands derived in [49]. This model establishes that the horizontal acceleration of the MAV is proportional to the horizontal force acting upon the quadcopter, that is, the accelerating force minus the drag force. The drag is proportional to the horizontal velocity of the quadcopter, while the accelerating force is proportional to a projection of the z-axis onto the horizontal plane, resulting in the following equations:

(7) $${\dot{vx}}_{t}=K_{1}\left(K_{2}\left(\cos\phi_{t}\sin\theta_{t}\cos\psi_{t}+\sin\phi_{t}\sin\psi_{t}\right)-vx_{t}\right)$$

(8) $${\dot{vy}}_{t}=K_{1}\left(K_{2}\left(\cos\phi_{t}\sin\theta_{t}\sin\psi_{t}-\sin\phi_{t}\cos\psi_{t}\right)-vy_{t}\right)$$

On the other hand, the influence of the sent control command $u=\left(\hat{vx},\hat{vy},\hat{vz},\;\hat{\dot{\psi }}\right)$ is described by the following linear model, taking into account the angle direction criterions shown in Figure 1:

(9) $${\dot{\phi }}_{t}=-K_{3}\left(K_{4}\hat{vy_{t}}+\phi_{t}\right)$$

(10) $${\dot{\theta }}_{t}=K_{3}\left(K_{4}\hat{vx_{t}}-\theta_{t}\right)$$

(11) $${\dot{vz}}_{t}=K_{5}\left(K_{6}\hat{vz_{t}}-vz_{t}\right)$$

(12) $${\ddot{\psi }}_{t}=K_{7}\left(K_{8}\hat{{\dot{\psi }}_{t}}-{\dot{\psi }}_{t}\right)$$

We estimated the proportional coefficients K_1_ to K_8_ for the different platforms from data collected in a series of test flights. From Equations (7) to (12) we obtain the following overall state transition function:

(13) $$\left(\begin{array}{c} x_{t+1}\\ y_{t+1}\\ z_{t+1}\\ vx_{t+1}\\ vy_{t+1}\\ vz_{t+1}\\ \phi_{t+1}\\ \theta_{t+1}\\ \psi_{t+1}\\ {\dot{\psi }}_{t+1} \end{array})⟵(\begin{array}{c} x_{t}\\ y_{t}\\ z_{t}\\ vx_{t}\\ vy_{t}\\ vz_{t}\\ \phi_{t}\\ \theta_{t}\\ \psi_{t}\\ {\dot{\psi }}_{t} \end{array})+\Delta_{t}(\begin{array}{c} vx_{t}\\ vy_{t}\\ vz_{t}\\ K_{1}\left(K_{2}\left(\cos\phi_{t}\sin\theta_{t}\cos\psi_{t}+\sin\phi_{t}\sin\psi_{t}\right)-vx_{t}\right)\\ K_{1}\left(K_{2}\left(\cos\phi_{t}\sin\theta_{t}\sin\psi_{t}-\sin\phi_{t}\cos\psi_{t}\right)-vy_{t}\right)\\ K_{5}\left(K_{6}\hat{vz_{t}}-vz_{t}\right)\\ -K_{3}\left(K_{4}\hat{vy_{t}}+\phi_{t}\right)\\ K_{3}\left(K_{4}\hat{vx_{t}}-\theta_{t}\right)\\ {\dot{\psi }}_{t}\\ K_{7}\left(K_{8}\hat{{\dot{\psi }}_{t}}-{\dot{\psi }}_{t}\right) \end{array}\right)$$

#### 3.3.2. Navigation Data Observation Model

This model relates the state vector with the onboard measurements obtained through the navigation channel of the MAV (that we called “Navdata” in Figure 3). According to the minimal onboard sensor configuration described in Section 2.2, this Navdata channel must at least provide the drone’s height $\bar{h}$ obtained from an altimeter (ultrasonic sensors in our both platforms) and the drone’s orientation as roll, pitch and yaw angles $\left(\bar{\phi },\bar{\theta ,}\bar{\psi }\right)$ obtained from the gyroscope. It results in the following minimal measurement vector:

(14) $$z_{NAVDATA,t}={\left({\bar{h}}_{t},{\bar{\phi }}_{t},{\bar{\theta }}_{t},{\bar{\psi }}_{t}\right)}^{T}$$

When other onboard measurements are available, this measurement vector can easily be enlarged in order to incorporate them. This is the case of the Bebop drone, which provides a measurement of the horizontal velocities of the drone $\left(\bar{vx},\bar{vy}\right)$ in the stabilized coordinate frame {S} obtained through the downfacing camera:

(15) $$z_{NAVDATA,t}={\left({\bar{h}}_{t},{\bar{\phi }}_{t},{\bar{\theta }}_{t},{\bar{\psi }}_{t},{\bar{vx}}_{t},{\bar{vy}}_{t}\right)}^{T}$$

The roll and pitch angles measured by the gyroscope can be considered as direct observations of the corresponding state variables. Furthermore, we differentiate the height and yaw measurements to be used as observations of their respective velocities. And if present, the velocity measurements of the downfacing camera have to be transformed from the stabilized coordinate frame {S} into the world frame {W}. The resulting measurement vector $z_{NAVDATA}$ and observation function $h_{NAVDATA}\left(\chi_{t}\right)$ are:

(16) $$z_{NAVDATA,t}={\left({\bar{h}}_{t}-{\bar{h}}_{t-1}+z_{t-1},{\bar{\phi }}_{t},{\bar{\theta }}_{t},{\bar{\psi }}_{t}-{\bar{\psi }}_{t-1}+\psi_{t-1},{\bar{vx}}_{t},{\bar{vy}}_{t}\right)}^{T}$$

(17) $$h_{NAVDATA}\left(\chi_{t}\right):=\left(\begin{array}{c} z_{t}\\ \phi_{t}\\ \theta_{t}\\ \psi_{t}\\ vx_{t}\cos\psi_{t}+vy_{t}\sin\psi_{t}\\ -vx_{t}\sin\psi_{t}+vy_{t}\cos\psi_{t} \end{array}\right)$$

#### 3.3.3. VSLAM Observation Model

When the VSLAM module successfully tracks a video frame, its 6-DoF pose estimation is transformed from the coordinate system of the front camera to the coordinate system of the drone {D}, leading to a direct observation of the drone’s pose given by:

(18) $$z_{VSLAM,t}=f\left(E_{DC}E_{C,t}\right)$$

(19) $$h_{VSLAM}\left(\chi_{t}\right):={\left(x_{t},y_{t},z_{t},\phi_{t},\theta_{t},\psi_{t}\right)}^{T}$$

where $E_{C,t}\;\epsilon \;SE\left(3\right)$ is the estimated camera pose, $E_{DC}\;\epsilon \;SE\left(3\right)$ the constant transformation from the camera to the quadcopter coordinate system and $f:SE\left(3\right)\cdot {ℜ}^{6}$ the transformation from an element of *SE*(3) to the position and roll-pitch-yaw representation.

#### 3.3.4. Scan Matcher Observation Model

The scan matcher obtains a 3-DoF estimation of the MAV’s footprint pose, that is considered as a direct observation of the corresponding state variables, as it is shown in the following linear observation model:

(20) $$z_{LASER,t}={\left({\bar{x}}_{t},{\bar{y}}_{t},{\bar{\psi }}_{t}\right)}^{T}$$

(21) $$h_{LASER}\left(\chi_{t}\right)\cdot {\left(x_{t},y_{t},\psi_{t}\right)}^{T}$$

### 3.4. PID Controller

Once the estimated position of the MAV is provided by the EKF, a PID controller has been designed to control de movements of the MAV. A reference $\left(\hat{x},\hat{y},\hat{z},\hat{\psi }\right)$ is needed as the desired position of the drone in world coordinates. The EKF will bring the estimation of the pose, as shown in Figure 3. The difference between the reference and the estimated pose is the error that will be minimized by the PID controller, by sending to the MAV an appropriate control command $u=\left(\hat{vx},\hat{vy},\hat{vz},\;\hat{\dot{\psi }}\right)$, that is calculated in the following way:

(22) $${\hat{vx}}_{t}=\cos\psi_{t}\left[Kp\left({\hat{x}}_{t}-x_{t}\right)+Kd\cdot {\dot{x}}_{t}\right]+\sin\psi_{t}\left[Kp\left({\hat{y}}_{t}-y_{t}\right)+Kd\cdot {\dot{y}}_{t}\right]$$

(23) $${\hat{vy}}_{t}=-\sin\psi_{t}\left[Kp\left({\hat{x}}_{t}-x_{t}\right)+Kd\cdot {\dot{x}}_{t}\right]+\cos\psi_{t}\left[Kp\left({\hat{y}}_{t}-y_{t}\right)+Kd\cdot {\dot{y}}_{t}\right]$$

(24) $${\hat{vz}}_{t}=Kp\cdot \left({\hat{z}}_{t}-z_{t}\right)+Kd\cdot {\dot{z}}_{t}+Ki\cdot \int \left({\hat{z}}_{t}-z_{t}\right)$$

(25) $${\hat{\dot{\psi }}}_{t}=Kp\left({\hat{\psi }}_{t}-\psi_{t}\right)$$

It allows the algorithm to drive the MAV along a series of points in the world coordinate frame {W}, so it can follow a specific trajectory, as will be shown in the results section.

### 3.5. Delay Compensation

For controlling a quickly reacting system such as a MAV, an accurate and delay-free state estimation is required. The delays in the estimation lead to a poor control even if the estimation is correct. When using a low-cost MAV, the delay caused by the wireless communication channel must be taken into account, mainly in the transmission of the compressed camera images. The time required between the instant a frame is captured and the instant the respective calculated control signal is applied (i.e., the time required for encoding the image on the drone, transmitting it via wireless LAN, decoding it on the PC, applying visual SLAM, data fusion and control calculations and transmitting the resulting control signal back to the drone) lies between 160 ms and 300 ms. Fortunately, these delays can be easily compensated within the EKF framework.

Firstly, we chose an execution period for our SLAM system that ensures that a new video frame would be processed on each iteration of the system. The VSLAM algorithm takes a median of 35 ms depending on the selected method, while the execution time of the scan matcher is about 10 ms. The EKF and PID controller execution times are much lower, of only a few milliseconds. So, an execution period of T = 50 ms has been chosen, so that *t_SLAM+PID_* < *T*.

The amount of time that (1) a new frame captured by the frontal camera needs to be transmitted via wireless LAN to the ground station *t_video_trans_* and (2) a calculated control command needs to reach the MAV and take effect *t_command_trans_*, depend on the bandwidth used by nearby wireless LAN networks. If the onboard processor of the MAV is open-access (as in the case of the Erle-Copter), these times can be estimated by measuring two latency values using an echo signal to the drone, and they can be updated in regular intervals while the connection is active. In our tests environment, *t_video_trans_* typically lies between 50 ms and 200 ms and *t_command_trans_* between 30 ms and 100 ms. In our system, these delays are rounded to an integer multiple of the SLAM execution period *T*, being:

(26) $$n=round\left(\frac{t_{video\_trans}}{T}\right)$$

(27) $$m=round\left(\frac{t_{command\_trans}}{T}\right)$$

As the measurements of the Navdata channel are transmitted each 5 ms, and the readings and processing of the scan matcher are performed in less than 10 ms, these delays are neglected because they are lower than the execution period *T*.

**Figure 7:**
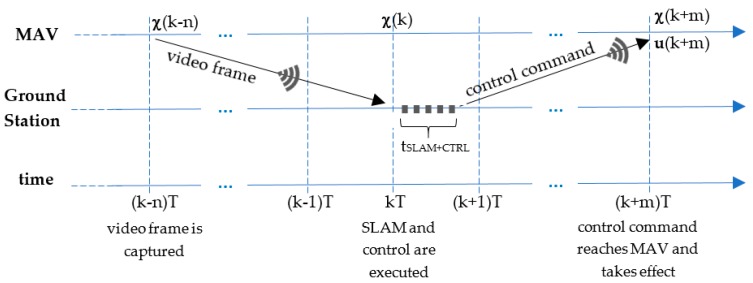
Times and delays involved in one iteration of the SLAM system at t = kT.

Figure 7 shows a time diagram with the main times and delays involved in one iteration of the SLAM system at time *t* = kT. The video frame processed by the VSLAM module at that time was captured by the drone in a time corresponding to n iterations before, and so it must be used to correct the estimation of the state $\chi_{t=\left(k-n\right)T}=\chi \left(k-n\right)$. After that, the EKF is rolled forward up to the current time *t* = kT, using the prediction stage with buffered previously sent commands and the correction stage with laser and navdata buffered previous measurements to estimate the current state $\chi_{t=kT}=\chi \left(k\right)$. Then, only the prediction stage is used to estimate the future pose of the MAV $\chi_{t=\left(k+m\right)T}=\chi \left(k+m\right)$ when the control command reaches the MAV at t=(k+m)T. This estimate is used to calculate the control command $u_{t=\left(k+m\right)T}=u\left(k+m\right)$ that is sent to the MAV and stored in the buffer for future predictions.

## 4. Experimental Results

In this section, we present the experimental results that validate our proposed SLAM system. Firstly, we show some practical aspects about our test bed that include the software implementation and the ground truth system used for validating the estimation results. After that, we show the experimental results obtained with the minimal configuration of the SLAM system that includes only VSLAM, IMU and an altimeter. Finally, some tests that include laser measurements are shown, comparing the effect of using the different sensors separately and fused in the proposed EKF.

### 4.1. Implementation and Ground Truth System

Two different low-cost commercial MAVs have been used in our experiments: the Bebop drone (Figure 2a) and the Erle-Copter (Figure 2b), whose main features were detailed in Section 2.2. It proves that the proposed SLAM system can be implemented in different commercial platforms with different sensory configurations, with only minor modifications. In order to achieve this scalability, a node-based software network has been developed based on the ROS framework, using well-tested packages when available.

**Figure 8:**
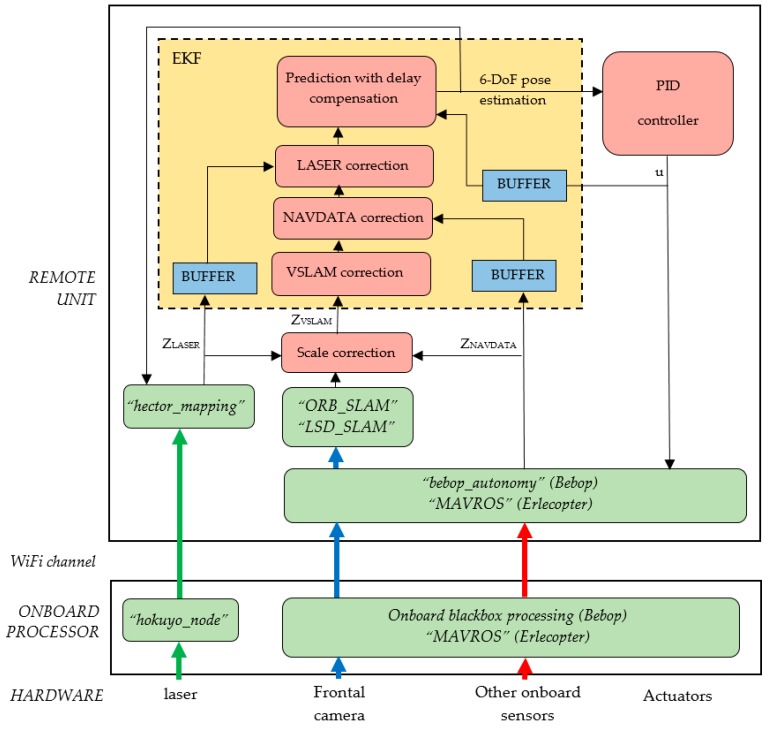
Software implementation of the SLAM system: green blocks correspond to a priori available ROS packages; custom blocks provided to the SLAM and control systems are shown in red.

Figure 8 shows this software implementation. Green blocks correspond to a priori available ROS packages: (a) “bebop_autonomy” [40] and “MAVROS” [42] for communicating with the autopilots of Bebop and Erle-Copter respectively, sending control commands and receiving onboard sensor data; (b) “hokuyo_node” [41] to provide access to the Hokuyo laser range finder; (c) “hector_mapping” as a part of the Hector-SLAM stack [64] to implement the scan matcher module; and (d) “ORB-SLAM” [65] or “LSD-SLAM” [66] as implementations of the two tested VSLAM methods. The custom blocks provided to the SLAM and control systems, also as ROS nodes, are shown in red color.

**Figure 9:**
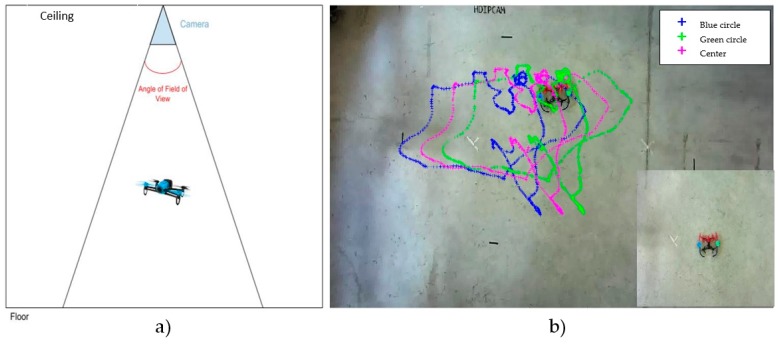
(**a**) Ground truth system based on a monocular camera on the ceiling; (**b**) Example of tracking of the MAV flight: blue crosses represent the locations of the blue marker, green crosses the green marker ones, and purple crosses the calculated center of the MAV.

One of the major difficulties of the experimental setup was to design a ground truth system to validate the pose estimation based on an external sensor. Since a motion capture system that could obtain a reliable measurement of the actual six degrees of freedom position of the MAV was not available, we have used a simplified system that allows us to approximately estimate some coordinates of the MAV’s pose under certain assumptions. The value of this ground system is that it has not accumulative error. It is based on a monocular camera on the ceiling of the test area, as it is shown in Figure 9a. Adding a pair of distinguishable artificial markers to the MAV (two coloured circles, as it is shown in Figure 9b), it is possible to estimate the (x,y,z) and ѱ coordinates of the MAV, using some basic geometric relationships under the assumption that the drone is always in an horizontal plane (which is quite realistic because we configured the drone to move slowly in the horizontal plane). Under this assumption, the distance between markers gives the height z of the MAV, and the x, y and ѱ coordinates can be easily extracted.

As a disadvantage of this ground truth system, it only covers an open test area of 5 × 5 m. This is sufficient for testing the SLAM system with the minimal sensor configuration (based on VSLAM and onboard sensors), by commanding some square trajectories to the control system and comparing the estimated trajectories with the ground truthed ones. However, this test environment (that we call “Ground-truthed test environment”) is not suitable to validate the SLAM system when using also laser measurements, because walls are so far to be detected with our laser sensor. For this reason, we have used two other different scenarios to validate the complete SLAM system, coinciding with the two environments in which we performed the initial tests of the VSLAM and scan matcher modules. These are the storage area shown in Figure 5 (called “Storage room test environment”) and the lab and corridors sequence shown in Figure 6 (called “Corridors test environment”). In these last environments we do not have a ground truth system, but we can use some metric measurements to validate trajectory estimations.

### 4.2. Results with the Minimal Sensor Configuration: VSLAM, IMU and Altimeter

In this section we include some experimental results obtained with the Bebop drone in the ground-truthed test environment. In this case we use the minimal sensor configuration of the proposed SLAM system that includes the horizontal camera, IMU and altimeter. The objective of these experiments is to compare the ability of LSD-SLAM and ORB-SLAM when they are applied to a MAV, by solving the scale ambiguity and improving the state estimation fusing the IMU and altimeter data in the EKF. In these tests, the PID controller receives a series of points that conform a rectangle of 120 cm × 60 cm as reference path, maintaining the reference orientation of the drone constant along this path.

**Figure 10:**
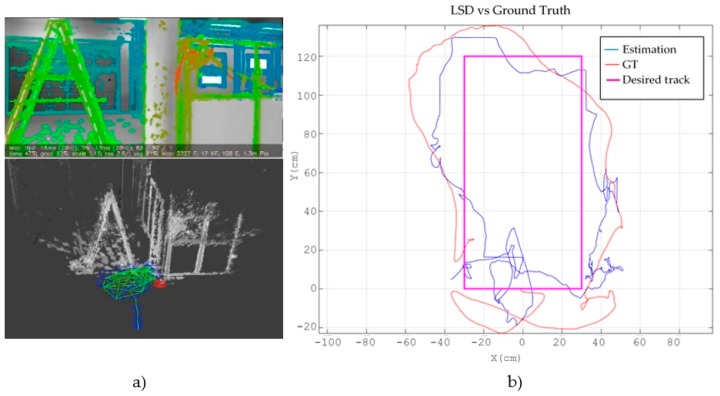
Results obtained with the minimal sensor configuration using LSD-SLAM. (**a**) shows the view of the frontal camera with the obtained depth map at the top and the estimated trajectory embedded in the cloud points map below; (**b**) shows the desired track projected in the XY plane (magenta colour), the ground truth path obtained with the external camera (blue colour) and the path estimated with the SLAM system (red colour).

Firstly, we show the results of using all the stages of the EKF with the two different VSLAM methods in order to compare them. Figure 10 shows the experiment with LSD-SLAM. At the left, Figure 10a displays the view of the frontal camera with the obtained depth map and, below it, the estimated trajectory embedded in the environment’s cloud points map. On the right, Figure 10b shows the desired track projected in the XY plane (magenta colour), the ground truth track obtained with the external camera (blue colour) and the path estimated with the SLAM system (red colour). It can be seen that, as expected in a fast dynamic system as a MAV, there are some deviations between the reference trajectory and the actual one that could be mitigated with more sophisticated control laws. However, the position estimated by the proposed SLAM system is good enough to control the MAV.

**Figure 11:**
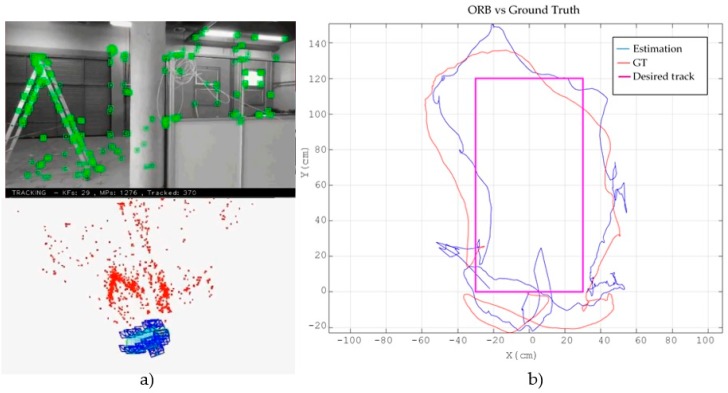
Results with the minimal sensor configuration using ORB-SLAM. (**a**) shows at the top the view of the frontal camera with the obtained marks and below, the points cloud map and the estimated trajectory; (**b**) shows the desired track projected in the XY plane (magenta colour), the ground truth path obtained with the external camera (blue colour) and the path estimated with the SLAM system (red colour).

Figure 11 shows the same experiment using ORB-SLAM. Figure 11a displays the obtained marks cloud that conform the map and the estimated trajectory, while Figure 11b shows the projection onto the XY plane of the desired (magenta), actual (red) and estimated (blue) trajectories.

In order to compare both VSLAM methods with our own sensor and ground truth data, we have calculated the same translational error measures that we obtained with the external benchmark and dataset used in Section 3.1.3. Five tests were made using each VSLAM algorithm, and median results are shown in Table 2. In addition to the results obtained in Section 3.1.3, these new tests confirm that ORB-SLAM is more accurate than LSD-SLAM in well-lit environments with sufficient visual features. Besides, the errors are smaller than those obtained when using only the VSLAM algorithm with scale correction, thanks to the prediction and correction stages of the EKF. Table 3 presents the error between the estimated yaw angle and the one obtained from the ground truth system using ORB-SLAM. It can be seen that this error is kept within close tolerances with this method. On the contrary, LSD-SLAM presents tracking problems with fast rotational movements, which leads us to choose ORB-SLAM as first choice in sufficiently illuminated and featured environments.

On the other hand, the main contribution of the proposed SLAM system is multi-sensor fusion, exploiting the typical onboard sensors of commercial drones. The usage of IMU and altimeter measurements, all them integrated into an EKF that also takes into account the commanded velocities through its prediction stage, greatly improves the estimation results of either of the VSLAM techniques.

**Figure 12:**
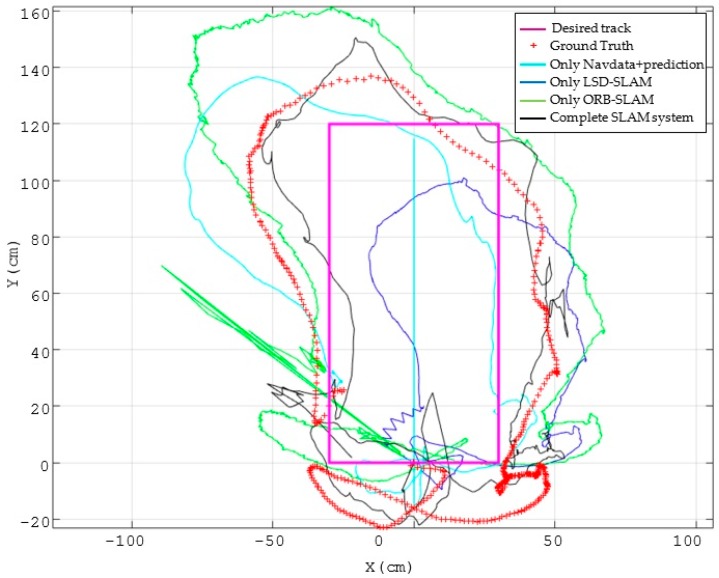
Results with different SLAM configurations: (1) using only proprioceptive sensors (Navdata) and the prediction in the EKF (light blue line); (2) using only VSLAM with scale correction, LSD-SLAM is shown in dark blue and ORB-SLAM in green; (3) fusing all the measurements and the prediction in the EKF (black line). Red crosses show the actual trajectory of the drone obtained with the ground truth system.

In Figure 12, red crosses show the actual trajectory of the drone when following the previously established reference points, obtained with the ground truth system. The light blue trajectory is the estimated position of the MAV using only the proprioceptive information of the drone, namely the Navdata measurements and the prediction model of the EKF. Since in this case no external reference is used to correct the estimation, a large deviation respect to the actual trajectory is addressed. In dark blue and green we show the results of both VSLAM techniques, LSD-SLAM and ORB-SLAM respectively, using the altimeter sensor only for scale correction.

In these cases, only the visual correction model is used in the EKF and we can see that the estimation results are very poor. Black line shows the estimated trajectory when fusing all the information in the EKF. It is easily observable that estimation results are clearly improved. Table 4 shows a comparison of the translational errors, demonstrating that they are drastically reduced to the half, getting an RMSE error of around 5 cm.

### 4.3. Results Including the Laser Sensor

In this section we show the experimental results obtained with the Erle-Copter drone using the complete multi-sensorial SLAM system (laser, vision, IMU and altimeter). As it was stated, these experiments have been performed in confined test environments in which walls can be detected by our URG-04LX sensor (Hokuyo, Osaka, Japan) whose detection range is 4 m. In these environments we do not have a ground truth system, so we use some metrics of the followed trajectory on the XY plane to validate the estimations.

**Figure 13:**
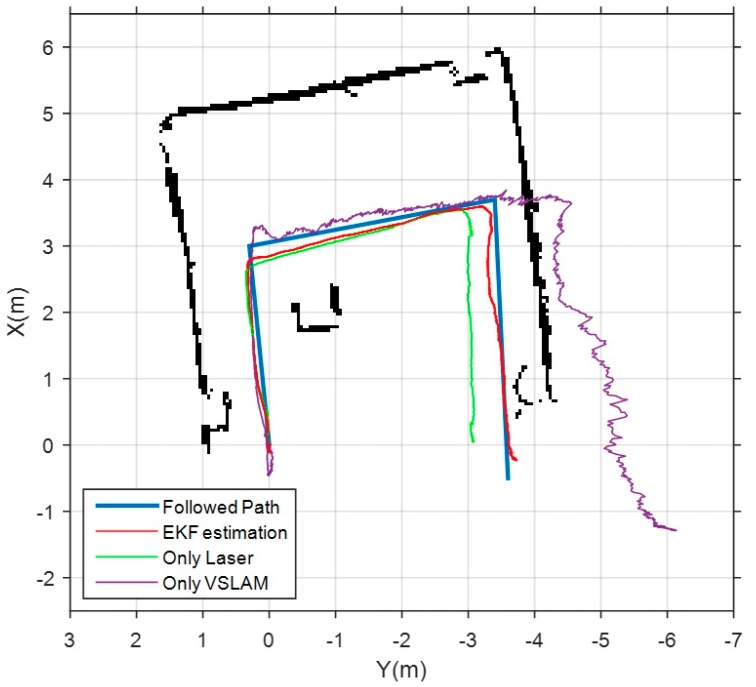
Results obtained in the “storage room test environment”. Blue line represents the metrics of the actual followed path. Magenta line shows the estimation of the VSLAM module. Green line corresponds to the estimation of the scan matcher module. Red line is the trajectory estimated by the complete SLAM system.

Firstly, the results obtained in the storage room test environment are shown in Figure 13. The blue line represents the metrics of the actual path followed by the drone. Three different estimations are shown in the same figure. The magenta trajectory corresponds to the estimation of the VSLAM module using ORB-SLAM. As previously noted, the poor lighting in this area yields to a wrong estimation of the length of the second and third segments of the path. The green trajectory shows the estimation performed by the scan matcher module. As it is a confined space with easy features as corners, this estimation is better than the visual one, but, even so, a shortening effect is observed. Finally, the red trajectory shows the estimation obtained as output of the EKF with our complete SLAM system. In this case, laser and visual estimations are fused with the measurements of IMU and altimeter, and combined with the prediction stage of the EKF. This results in a much more accurate pose estimation, as it is shown in Table 5, that displays the translational errors of the three estimations.

**Figure 14:**
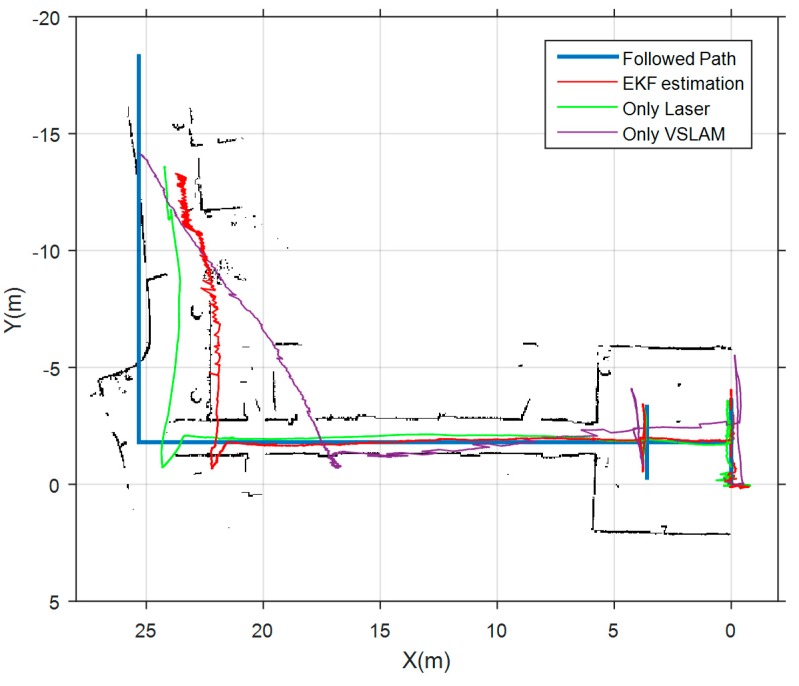
Results obtained in the “corridors test environment”. Blue line represents the metrics of the followed path. Magenta line shows the estimation of the VSLAM module. Green line corresponds to the estimation of the scan matcher. Red line is the trajectory estimated by the complete SLAM system.

Finally, we show in Figure 14 the results obtained in the corridors test environment. In this case, the followed path starts in a well-lit room, and continues along two L-shaped corridors in which nearly all doors were closed, hindering laser features detection. Again, blue line is the actual followed path, magenta and green lines are the estimations of the VSLAM module and scan matcher module respectively. The red path is the estimation of our complete SLAM system. The three estimations provide good results in the initial room, because it is a well-lit confined area. However, it is observed that both laser and vision produce a shortening effect in the estimation of the length of the corridors. This effect is also reflected in the laser map that is constructed by the scan matcher module using only laser measurements. Although the estimation of the laser module is much more accurate, it cannot compensate the harmful effect of VSLAM in the corridor. So, translational errors become quite large in this environment, as can be verified in Table 6. Anyway, the scan matcher module allows a clear improvement in the results of the estimation. This experiment proves that pose estimation along corridors continue to be a challenge in indoor navigation of aerial robots that do not dispose of classical odometric systems based on direct contact with the environment.

Taking these results into account, the authors of this paper are currently working on a module that automatically detects and classifies the environment using different visual and structural descriptors (“corridor”, ”hall”, ”well-lit”, ”poorly-lit”, etc.). This classifier will use the onboard sensors of the MAV (laser and vision) as source information. The result of the classification will be used to automatically adapt the SLAM system parameters to obtain the best results in each situation. For example, in corridor environments could be useful to compensate the shortening effect of both main sensors (laser and vision) by overestimating the movement of the MAV in the prediction stage of the EKF, and/or increasing the covariance matrixes of laser and vision observation models. Indeed, it may be desirable to completely discard VSLAM in poorly lit environments or the scan matcher estimation in wide environments out of the detection range of the laser sensor.

## 5. Discussion

Estimating the movement of a MAV relative to its environment is a hard challenge. In indoor and GPS-denied environments, systems that achieve similar results to classical odometric methods for ground robots have not been yet developed.

The SLAM system proposed in this paper has to be evaluated in the context of the application to which it is intended. As we stated in the introduction, a small low-cost MAV with very limited onboard computational capacity moves near a ground robot with a powerful remote unit which executes the SLAM algorithms. So, execution time is not a constraint in this case. However, only typical onboard sensors can be used and communication delays must be taken into account.

One of the requirements imposed to our system is sensor configurability, that we achieve using an EKF for sensor fusion. A minimal sensor configuration that adds the monocular camera to typical proprioceptive sensors (IMU and altimeter) has been supposed. We have demonstrated that state-of-the-art monocular VSLAM methods can be applied to aerial robots taking advantage of onboard sensors to solve scale ambiguity and to improve the pose estimation through the EKF. So far, PTAM has been the most popular choice for implementing VSLAM in aerial robots. In this work, we have compared two more recent and precise methods as ORB-SLAM and LSD-SLAM, proposing a scale correction method based on altimeter and laser (if available) measurements. ORB-SLAM has demonstrated to be a more accurate method in visually positive environments, such as well-lit and sufficiently featured ones. It also provides better results with rotational movements, while LSD-SLAM could be a better choice in featureless environments. In any case, our SLAM proposal fuses the estimate of VSLAM with the onboard sensor measurements and a prediction model, and this is proven to provide a substantial improvement in the pose estimation. As a future work line, the VSLAM method could be automatically chosen as a function of the characteristics of the environment and the trajectory.

When available, a 2D laser rangefinder is a highly suited sensor for MAVs in indoor environments, due to its fast and direct range detection, so a scan matcher module has been added to our EKF-based SLAM framework, based on the Hector Mapping algorithm. It has been demonstrated that in most environments, the fusion of visual and laser information with onboard sensors and prediction models provides a better estimation result. However, some environments as corridors are still a major challenge because both sensors (vision and laser) tend to short their length. Although this is compensated with IMU measurements and the prediction model, estimation results are still poorer than in more featured environments. As a future research, we are working on detecting and classifying the environment into some types to adjust the covariance matrixes of the EKF to minimize these undesired effects.

It is important to note that we use SLAM as a framework for robust pose estimation and tracking. Obtaining a global and consistent map is not within the objectives of this work. So, depth maps or point clouds are used only as local maps for localization, and discarded once the MAV obtains a new keyframe. Furthermore, loop detection and closure methods have not been added to our system. As a future research line, we are working on integrating visual 3D maps with laser 2.5D maps to obtain wide global and consistent maps.

Therefore, the importance and contributions of this paper are focused on the fusion of several sensors for solving the SLAM problem of a complex robot platform as a MAV. We have applied successful state-of-the-art algorithms in order to advance the research in the fusion and control topics. We have developed specific models for fusing the estimations of all the sensors of the MAV (vision, laser, altimeter and IMU) and a prediction model to improve the estimates with respect to those made independently by any of the sensors. We have demonstrated that the proposed SLAM architecture can be used in real time for controlling the movements of the MAV.

## 6. Conclusions

This paper shows a multi-sensorial SLAM system that successfully tracks the 6-DoF pose of low-cost MAVs in GPS-denied environments using a remote control station. To do this, several state-of-the art SLAM algorithms have been applied and compared. The fusion of monocular vision with laser measurements (when available) and other typical onboard sensors (IMU and altimeter) by means of an EKF framework simplifies the configurability of the system. Using two commercial platforms, it has been demonstrated with experimental results that the proposed SLAM system improves the results of the baseline techniques when estimating the trajectory of the MAV in different environment conditions.

## Figures and Tables

**Table 1:** Absolute translational error comparison between both monocular VSLAM algorithms using the “RGB-D SLAM Dataset and Benchmark” of the Computer Vision Group from Technische Universität München [56].

	LSD-SLAM	ORB-SLAM
Compared pose pairs	782	283
RMSE ^1^	8.09 cm	6.9 cm
Mean	6.74 cm	5.10 cm
Median	5.57 cm	3.85 cm
Standard Deviation	5.82 cm	4.61 cm
Min	3.30 cm	0.23 cm
Max	27.92 cm	26.08 cm

^1^ RMSE: Root-mean-square deviation.

**Table 2:** Absolute translational error comparison between both monocular VSLAM algorithms using the complete SLAM system with the minimal sensor configuration.

	LSD-SLAM	ORB-SLAM
Compared pose pairs	210	348
RMSE	6.74 cm	5.32 cm
Mean	4.83 cm	3.90 cm
Median	3.21 cm	2.79 cm
Standard Deviation	4.69 cm	3.61 cm
Min	0.22 cm	0.61 cm
Max	18.23 cm	14.52 cm

**Table 3:** Absolute yaw error using ORB-SLAM in the complete SLAM system with the minimal sensor configuration.

	ORB-SLAM
Compared pose pairs	348
RMSE	6.53°
Mean	5.34°
Median	4.88°
Standard Deviation	0.60°
Min	2.53 × 10^−5^°
Max	15.37°

**Table 4:** Translational errors in the “ground-truthed test environment”.

	Only Navdata and Prediction	Only VSLAM (ORB-SLAM)	Complete SLAM System
Compared pose pairs	332	158	332
RMSE	11.52 cm	10.71 cm	5.32 cm
Mean	9.42 cm	8.49 cm	3.97 cm
Median	8.78 cm	6.87 cm	3.05 cm
Standard Deviation	6.63 cm	6.52 cm	3.54 cm
Min	0.08 cm	0.12 cm	0.01 cm
Max	23.49 cm	29.89 cm	14.82 cm

**Table 5:** Translational errors in the “storage room test environment”.

	Only Laser (Hector-Mapping)	Only VSLAM (ORB-SLAM)	Complete SLAM System
Compared pose pairs	206	156	287
RMSE	23.81 cm	28.85 cm	9.44 cm
Mean	17.84 cm	12.71 cm	7.82 cm
Median	10.31 cm	6.60 cm	7.81 cm
Standard Deviation	15.76 cm	25.90 cm	5.30 cm
Min	0.26 cm	0.03 cm	0.03 cm
Max	61.82 cm	154.14 cm	25.88 cm

**Table 6:** Translational errors in the “corridors test environment”.

	Only Laser (Hector-Mapping)	Only VSLAM (ORB-SLAM)	Complete SLAM System
Compared pose pairs	104	88	93
RMSE	73.71 cm	179.38 cm	103.71 cm
Mean	46.73 cm	103.86 cm	49.82 cm
Median	16.82 cm	40.87 cm	10.73 cm
Standard Deviation	57.00 cm	146.25 cm	90.96 cm
Min	2.07 cm	0.88 cm	0.10 cm
Max	175.6 cm	615.80 cm	332.63 cm
